# Does inclusive leadership foster employee psychological resilience? The role of perceived insider status and supportive organizational climate

**DOI:** 10.3389/fpsyg.2023.1127780

**Published:** 2023-03-15

**Authors:** Li Xintian, Peng Peng

**Affiliations:** ^1^School of Business, Shandong Jianzhu University, Jinan, Shandong, China; ^2^School of Business, Qingdao University of Technology, Qingdao, China

**Keywords:** inclusive leadership, employee psychological resilience, supportive organizational climate, perceived insider status, social identity theory

## Abstract

**Introduction:**

Employee psychological resilience correlates with individual performance and well-being, which can help employees cope with work pressure under a complex situation. Drawing upon social identity and information processing theories, this paper explores how inclusive leadership stimulates employees’ psychological resilience by integrating the cross-level mediation effect of perceived insider status. This study scrutinized the moderating function of supportive organizational climate with inclusive leadership and employees’ perceived insider status, which expanded the inclusive leadership influence boundary.

**Methods:**

This study used a cross-sectional survey design and collected two-wave data from individuals who are currently employed in the context of Chinese organizations. Multiple linear regression was used to analyze the paired survey data of 220 employees of valid samples.

**Results:**

Inclusive leadership was positively related to employee psychological resilience; Perceived insider status mediated the relationship between inclusive leadership and employee psychological resilience; The indirect relationship above is moderated by supportive organizational climate such that the positive relationship will be enhanced when the supportive organizational climate is high, rather than low.

**Discussion:**

The theoretical and practical implications of these findings are discussed.

## 1. Introduction

The COVID-19 pandemic has brought a threat to life and a psychological impact on the public. Disruption of the pace of life and work in the context of the pandemic can easily lead to emptiness, depression, complaints and even anger among employees, reduce their psychological resilience and pessimistic expectations for the future, and let individuals take a negative approach to deal with work (Verdolini et al., 2021). These uncertainty and sudden changes undoubtedly increase employees’ anxiety and pressure, hinder employees’ perception of performance and psychological well-being, and even endanger the function and survival of the organization (Gimenez et al., 2017). Research has found that psychological resilience can help organizational members successfully resist risks, overcome adversity, quickly restore balance, and even achieve growth and development. It is regarded as an ideal trait that helps organizations and their members cope with multiple adversities (Linnenluecke, 2017). For managers, improving employees’ psychological resilience could reduce turnover and enhance performance (Dai et al., 2019). Thus, the research on the psychological resilience of employees has aroused widespread concern in academic and practical circles (Kantur and Say, 2015; Gimenez et al., 2017; Kuntz et al., 2017; Caniëls and Baaten, 2019).

According to the path of resilience development, employee psychological resilience (EPR) is a valuable psychological resource (Shin et al., 2012), which directly comes from individual resources and is supported and supplemented by the organizational environment (Kuntz et al., 2017). EPR is a critical capacity that results from the interaction between individual protective and environmental stimuli factors (e.g., adversity). Scholars explored the factors that supplement individual resources and stimulate EPR from organizational resources (Solberg and Wong, 2016), organizational environment (Kantur and Say, 2015), organizational climate (Caniëls et al., 2022), leadership characteristics (Kuntz et al., 2016) and other aspects. Most of these studies were based on the perspective of demand levels from individual resources (Luthans et al., 2010; Shin et al., 2012), but few studies focused on the antecedents and effect mechanism of EPR from the perspective of social identity theory and social information processing theory.

Leadership style is the situational factor that has the greatest impact on the behavior of subordinates (Owens and Hekman, 2016). Leadership style can effectively guide the behavior of employees and is seen as an essential trigger factor for psychological resilience (Zhu et al., 2019; Franken et al., 2020; Mao et al., 2022). When a leader has the right leadership style, people can develop psychological resilience, which can help them recover from stressful situations (Franken et al., 2020). Inclusive leadership (IL) is a kind of relational leadership (Nembhard and Edmondson, 2006) that has unique advantages in dealing with challenging environments. IL can gain insight into employees’ stress and emotional consumption which can be a superior option for increasing employees’ potential for psychological resilience (Li and Peng, 2022). Therefore, the direct impact of IL on EPR will be the first issue discussed in this paper. However, the mechanism of leadership style on EPR is not a simple stimulus to psychological capital, and there may be complex psychological processes between them.

Masterson and Stamper (2003) use the concept of perceived insider status (PIS) to describe an individual’s perception of the personal space, status, and acceptance he or she has earned within the organization as a member of the organization. Insider status is an immediate outcome of leader behavior, which eventually impacts employee behaviors in the workplace (Masterson and Stamper, 2003; Meng et al., 2019). Inclusive leaders embrace employee perspectives and misbehavior to facilitate the development of employee-owned ideas and suggestions, inspire higher levels of enthusiasm and motivation, and lead to higher task performance. By employing such inclusive approaches, managers encourage staff to accept diversity and provide staff with a sense of belonging in the workplace (Stamper and Masterson, 2002; Ahmed et al., 2020). Moreover, little is known about what makes employees consider themselves to be organizational insiders versus outsiders or the consequences of such a distinction between employees. According to social identity theory (Hogg, 2016), an increased perception of insider identity will help employees recognize their role expectations as organizational insiders and adjust their work attitudes and behaviors accordingly. Once employees are aware of “insider” status, they tend to show more psychological resilience and positive work performance, such as being more engaged and proactive in their work. We suggest that IL may indirectly affect EPR by mediating through PIS. Therefore, the second issue discussed in this paper is the mediating mechanism of PIS in the process of evoking EPR by inclusive leaders.

On the other hand, the effectiveness of leadership style depends on the organizational context (Lord et al., 2001), such as the organizational climate (Hayat and Afshari, 2021). The supportive organizational climate (SOC) originates from employees’ perceptions of their work content states and influences their social information processing processes and outcomes. It is a practical perspective for exploring organizational employees’ professional psychology and behavioral responses. It helps to clarify the boundaries of the role of inclusive leaders in contextualized research. Therefore, the third purpose is to explore the moderating role of SOC in the relationship between IL and EPR. Accordingly, this paper constructs and validates a moderated mediating effect model across levels to clarify the permeating effects, mediating paths, and boundary conditions of inclusive leaders on employee psychological resilience and attempts to propose a path for EPR cultivation and intervention to benefit the management practice of EPR.

## 2. Literature review and hypotheses development

### 2.1. Inclusive leadership and employee psychological resilience

The concept of inclusive leadership was first introduced by Nembhard and Edmondson (2006) to address the problems associated with employee diversity. IL is a leadership style that is good at listening to the views and recognizing the contributions of subordinates. Based on this definition, Carmeli et al. (2010) added IL as a core form of “relational leadership” and developed three dimensions to measure inclusive leadership: openness, accessibility, and availability. Inclusive leaders respect and acknowledge employees, focus on the needs and interests of employees, work with employees to accomplish tasks, and inspire more potential and energy (Hollander, 2009). Compared to other traditional leadership styles, IL is people-centered, better able to integrate people and issues, adapt to the complexity of management, and has its unique advantages in complex and changing management situations. IL can meet the needs of subordinates for commonality and differences at the same time, i.e., it is inclusive, open, democratic, and equal to each member, and it can accommodate the individual characteristics of subordinates and recognize the various demands and include all members in the organizational growth process. Compared to leadership forms such as charismatic leaders, transformational leaders, and humble leadership, IL styles emphasize more on the interaction and dependency between leaders and employees and have a profound driving effect on employees’ psychological capital (Javed et al., 2018; Li and Peng, 2022).

EPR is conceptualized as an “employee capability, facilitated and supported by the organization, to utilize resources to continually adapt and flourish at work, even if when faced with challenging circumstances.” EPR is the behavioral ability to use work resources to achieve continuous adaptation, physical and mental comfort, and growth of individual employees at work (Luthans et al., 2010). EPR emphasizes employees’ management, integration, and utilization of resources, which comprises a suite of behavioral characteristics: adaptability, learning, and network utilization (Kuntz et al., 2017). Like a proactive personality, psychological hardiness prompts resource utilization and is therefore expected to facilitate employee psychological resilience when support from the organization is provided. Individuals with high psychological resilience are more able to withstand stress than those with low psychological resilience, which further causes them to make different behavioral choices when facing stress. The greater the psychological resilience, the more positive emotions can be called upon, the higher the self-efficacy, the stronger the ability to resist psychological stress, and the weaker the opposite. Employee psychological resilience presented has practical utility to organizations; it makes it possible to assess the effectiveness of psychological resilience-building activities and identifies organization and personnel behaviors that need improvement. Hence, improving psychological resilience must take into account changes to organizational and intrapersonal elements that enable EPR development (Vanhove et al., 2016).

Previous scholars have suggested that leadership type is one of the key factors influencing the generation and development of EPR based on an integrated systems perspective (Zhu et al., 2019; Eliot, 2020). EPR can be attained by showing greater appreciation for employees’ contributions and being more receptive to novel ideas, which go beyond conventional psychological resilience-building techniques (Bardoel et al., 2014; Britt et al., 2016; Zhu et al., 2019). Leaders with a high level of psychological resilience can respond positively to crises that their organizations may face, and by demonstrating that psychological resilience and those positive responses, they can increase the psychological resilience of their employees. Psychological resilience can be developed in the presence of proper leadership and help employees recover from adversity (Franken et al., 2020). In line with the principle of reciprocity and social exchange theory, Mao et al. (2022) discovered that authentic leadership was significantly related to employee psychological resilience. Eliot (2020) explored that servant leaders focus their efforts on meeting their followers’ psychological needs and are thus well positioned to positively impact subordinate psychological resilience (Eliot, 2020). Zhu et al. (2019) explored that employees’ work-related promotion focus and perceived insider identity increased because of humble leadership, resulting in greater employee psychological resilience. These findings illuminate leadership as a new activator of employee psychological resilience that can be developed. Inclusive leaders are more likely to motivate and inspire their followers through enhanced internal stimulation, intrinsic zeal, and exposure to positive emotions like hope and optimism (Javed et al., 2017). In this regard, IL is helpful in assisting staff members in developing their capacity for psychological resilience and psychological resilience, making IL a better option for staff members’ ability for psychological resilience to increase. Employees will feel safer and consider their position more important when they believe their leaders are inclusive. As a result, they will display more psychological resilience by participating in and overcoming difficulties posed to their businesses. Therefore, we predict that IL is an antecedent of EPR. Hence, the following assumption is proposed:

*H1*: Inclusive leadership exerts a significant positive effect on employee psychological resilience.

### 2.2. The mediating role of perceived insider status

PIS reflects how included employees feel about inclusion in their firms (Stamper and Masterson, 2002). When an organization’s socializing methods and perks communicate to its employees that they have attained the status of an in-group member, a perspective like this one emerges (Lapalme et al., 2009). According to social identity theory, the way an organization treats an individual significantly influences the attitude and behavior of that individual while at work because it conveys an identity-related message to the employee (Brown, 2000). When an individual feels appreciated and valued by the organization, it means that he or she is respected and enjoys a higher status or position in the organization (Brown, 2000). Researchers have argued that individuals’ perceptions of their status in the organization, as well as the socio-emotional needs of self-esteem, belonging, and attachment, help them in incorporating their position status and organizational membership into their self-concept, thus enhancing their self-identity and thus contributing to the enhancement of employees’ psychological capital. Employees with high insider status are therefore more likely to accept responsibilities as organizational citizens, to have positive organizational attitudes, affective commitment, intention to stay, and behavior that supports organizational functions (Stamper and Masterson, 2002; Xiong Chen and Aryee, 2007; Lapalme et al., 2009). Inclusive leaders help to increase the individual’s perception of insider identity and organization-based self-esteem, which in turn generates a higher level of identification and psychological capital with the organization. In other words, inclusive leadership may inspire the critical perception that “I am an insider,” which stimulates the employee to further get psychological resilience. Integrating the past research findings with our current proposals, we will determine the path on that PIS mediates the relationship between IL and EPR capacity. Hence, the following assumption is proposed:

*H2*: Perceived insider status will mediate the relationship between inclusive leadership and employee psychological resilience.

### 2.3. The moderating effect of supportive organizational climate

Supportive organizational climate is defined by (Luthans et al., 2008) as the overall perception of employees’ support from colleagues and leaders and help from other departments and as members’ perceptions of the existence of a shared belief or culture in the organization that accepts or encourages certain behaviors (e.g., constructs) from members. Organizational support theory emphasizes that organizational support for employees is an important reason why employees are willing to stay and contribute to the organization, i.e., there is organizational support for employees before there is employee loyalty to the organization. Social information processing theory (Fulk et al., 1987) suggests that humans are adaptive organisms that understand and interpret their behavior and that of others based on the environment and adjust their attitudes and behaviors based on the information they receive. Social information processing theory is often used to explain the influence of leadership behavior and individual behavior on the formation of organizational climate or to explain the mechanism of organizational climate on employee behavior (e.g., suggestion behavior, helping behavior, etc.) and organizational outcomes (Priesemuth et al., 2014). According to information processing theory, an individual’s behavior is influenced by the work environment and the results of past behavior. Organizational climate, as information provided by the work environment, emerges from the social interactions of organizational members and influences employees’ attitudes and behaviors by providing cues and information about the organization’s desired, encouraged, and supported behaviors and develops in the process. Organizations with high levels of support encourage communication and information sharing, provide emotional support, etc., and promote a stronger perception of insider identity. It follows that inclusive leaders facilitate employees’ insider identity perceptions differently in different levels of supportive organizational climates. A supportive organizational climate influences employees’ social information processing processes and outcomes, adjusting the boundaries of the role of inclusive leaders. In a high SOC, the organization provides resources to support employees in completing their work tasks. Due to value heterogeneity, it compensates for the lack of emotional, informational, and psychological resources. At this time, individuals use these resources to gain emotional support, perceived self-efficacy, and autonomous control over their work and are more motivated to produce psychological resilience. Based on the above analysis, this paper proposes the hypothesis:

*H3*: Supportive organizational climate moderates the effect of inclusive leadership on employee psychological resilience.

*H4*: Supportive organizational climate moderates the mediating effect of perceived insider status in the relationship between inclusive leadership and employee psychological resilience.

**Figure 1:**
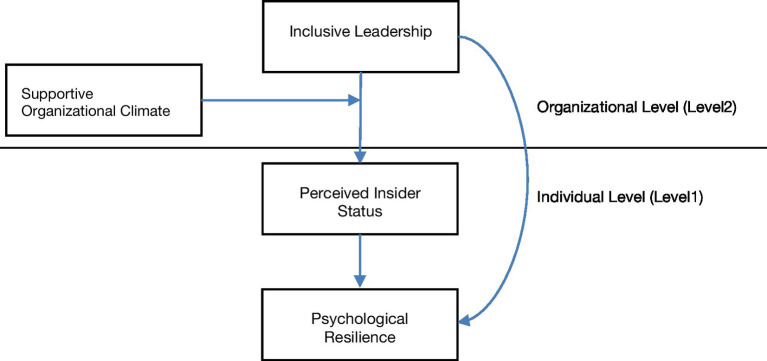
Theoretic analysis framework.

Based on the previous analysis, the following theoretical model is constructed in this study (see Figure 1).

## 3. Methods

### 3.1. Sample and process

This study used a questionnaire survey to obtain cross-sectional data, which focused on the period between September 2022 and October 2022, and the respondents were mainly from several service-oriented enterprises in finance, tourism, hotels, and restaurants in the eastern region of mainland China. Due to the COVID-19 epidemic, many enterprises did not welcome visits. We contacted the contacts of relevant enterprises through MBA alumni and other channels and explained to them the purpose of the study and related data collection. With their help, we adopted an online survey for data collection. To reduce the interference of social permissibility, we emphasized to all subjects before conducting the questionnaire that all questions in this study are not right or wrong, and the results of the survey are for research purposes only, and we assured that we would not disclose the results of the survey and personal information and asked the subjects to answer frankly. To reduce the possible homogeneous variance, we adopted two waves of the survey with a one-month interval. We matched the data by setting a four-digit code in questionnaire 1 and asking subjects to record and enter this code in questionnaire 2 to respond. To encourage subjects to answer, we added a bonus packet (WeChat red packet, a widely used electronic payment method in China) to the completion page of the electronic questionnaire, and a random bonus packet of 1–5 RMB could be drawn upon completion of the questionnaire. At time point T1, questionnaire 1 was administered to frontline employees of the above companies: it mainly included employee demographic information, inclusive leaders, supportive organizational climate, and other scales, and was collected; at time point T2, 1 month later, questionnaire 2 was administered to employees who had completed the questionnaire at time point T1: it mainly investigated employees’ perceived insider status and employee psychological resilience. After eliminating invalid and mismatched questionnaires, 220 valid matching questionnaires were used for the analysis. The results of the demographic data analysis of the study sample are as follows: In terms of gender, 80 were males (36.4%), and 140 were females (63.6%). In terms of age, 32 were below 24 years old (14.5%), 64 were between 25 and 30 years old (29.1%), 112 were between 31 and 40 years old (50.9%), and 12 were over 40 years old (5.5%). In terms of education, 22 were junior college (10.0%), 128 were in bachelor’s (58.2%), and 70 were in master’s (31.8%). The average organizational tenure is 3.4 years.

### 3.2. Measures

To ensure the reliability and validity of the measures, this study adopted the well-established scales widely used in the literature and translated them into Chinese using translation and back-translation procedures. IL, SOC, PIS, and EPR were all measured using Likert’s 5-point scale (1 = completely disagree, 5 = completely agree).

### 3.3. Inclusive leadership

We used a three-dimensional, 9-item scale developed by Carmeli et al. (2010) to measure IL. The scale has 3 sub-dimensions: openness, availability, and accessibility, Sample items were “managers are willing to listen,” “managers are willing to provide advice on work-related issues,” etc. The Cronbach’s alpha coefficients for the three sub-dimensions of inclusive leaders were 0.819, 0.861, and 0.847, respectively, and the Cronbach’s alpha coefficient for the overall scale was 0.896. Based on the theoretical hypotheses of this paper, and comparing the validity indicators, IL was examined as one dimension in this paper.

### 3.4. Perceived insider status

PIS was measured using a 6-item measure developed by Stamper and Masterson (2002). Sample items were “I feel very much a part of work organization” and “I feel I am an insider in this work organization.” The Cronbach α for the scale was 0.889.

### 3.5. Supportive organizational climate

The scale is based on the SOC Inventory developed by Luthans (Luthans et al., 2008) and revised to consider the actual situation of Chinese enterprises. This shortened scale contained aspects of climate most relevant to this study. Sample items were “I have a good working relationship with my manager,” and “Managers communicate work objectives and responsibilities.” The Cronbach α for the scale was 0.856.

### 3.6. Employee psychological resilience

EPR was measured using a 9-item measure by Nguyen et al. (2016). This measure aligns with our focus on psychological resilience as a skill-oriented construct focused on flexibility, problem-solving skills, and relationships as core dimensions. Sample item: “I use change at work as an opportunity for growth.” The Cronbach α for the scale was 0.938.

#### 3.6.1. Control variables

We used gender, age, education level, and organizational tenure as control variables in this study due to their potential and plausible influence on the outcomes.

### 3.7. Methodology

We used SPSS26.0 and AMOS26.0 for statistical analysis. First, descriptive analysis, reliability, and correlation analysis were performed using SPSS26.0. Second, Harmon one-way test and validated factor analysis were conducted using Amos 26.0 to test the common method bias and discriminant validity of the scales; Third, a multilayer linear model was constructed using SPSS 26.0 to test the hypothesis that and robustness testing of mediating effects using the Bootstrap method. Finally, the model with the SPSS macro program PROCESS was used to test the moderation mediating effect.

## 4. Results

### 4.1. Common method variance

Harman single-factor analysis was used to test the measurement model, and the results showed that no single factor was separated. The first factor only explained 28.53% of all measurement variations. It shows that this study has no serious problem of common method deviation. Thus, the common deviation had a limited effect in this study.

### 4.2. Descriptive statistical and correlations

Table 1 shows the descriptive statistics of all variables, mainly including mean, SD, minimum maximum, skewness, and kurtosis, which present the distributive qualities of these scales.

**Table 1:** Descriptive statistics.

Variable	*N*	Minimum	Maximum	Mean	SD	Skewness		Kurtosis	
	Statistic	Statistic	Statistic	Statistic	Statistic	Statistic	Std. error	Statistic	Std. error
Gender	220	0	1	0.36	0.482	0.571	0.164	−1.69	0.327
Age	220	1	4	2.47	0.808	−0.436	0.164	−0.53	0.327
Edu	220	2	4	3.22	0.610	−0.155	0.164	−0.505	0.327
Tenure	220	1	5	3.43	1.526	−0.375	0.164	−1.381	0.327
IL	220	1	5	3.56	0.811	−0.174	0.164	−0.179	0.327
PIS	220	1	5	3.28	0.881	−0.03	0.164	0.087	0.327
SOC	220	1	5	3.81	0.696	−0.132	0.164	−0.683	0.327
EPR	220	2	5	3.89	0.736	0.411	0.164	−0.879	0.327
Valid N	220	-	-	-	-	-	-	-	-

Gender (0 = female, 1 = male); *N* = 220.

Table 2 shows the correlations in which IL related positively to employee psychological resilience (*r* = 0.268, *p* < 0.01). IL also related positively to PIS (*r* = 0.569, *p* < 0.01), and PIS linked to EPR (*r* = 0.328, *p* < 0.01). All the correlations were in the predicted directions, which provided preliminary data support for the subsequent tests. The average variance extracted (AVE) values of the variables were also calculated in this paper, and the results are shown in Table 2, indicating that the average variance extracted (AVE) of the variables of the model reached an acceptable level (critical values: CR > 0.6; AVE > 0.5).

**Table 2:** Correlations for the study variables.

Variable	Mean	SD	CR	1	2	3	4	5	6	7	8
1.Gender	0.36	0.482	-	1							
2.Age	2.47	0.808	-	0.330**	1						
3.Edu	3.22	0.610	-	−0.128**	0.012	1					
4.Tenure	3.43	1.526	-	0.334**	0.665**	0.042	1				
5.IL	3.56	0.811	0.899	0.049	−0.241**	−0.085	−0.173**	(0.643)			
6.PIS	3.28	0.881	0.659	0.099	−0.177**	−0.043	−0.071	0.569**	(0.659)		
7.SOC	3.81	0.696	0.815	0.214	0.121	−0.004	0.103	0.284**	0.308**	(0.531)	
8.EPR	3.89	0.736	0.994	0.085	−0.053	0.008	0.090	0.268**	0.328**	0.629**	(0.773)

*p < 0.05; **p < 0.01; gender (0 = female, 1 = male); Numbers in diagonal brackets represent CR values; *N* = 220.

### 4.3. Confirmatory factor analysis

AMOS 26.0 was used to conduct Confirmatory Factor Analyses (CFA) on the research variables to test the differential validity of the variables. The four-factor model, three-factor model, two-factor model, and single-factor model are compared. The results show that the four-factor model is relatively well matched with the data [*χ*^2^/df = 1.384, CFI = 0.916, TLI = 0.922, and RMSEA = 0.045]. The fitting index between the four-factor model and the data is significantly better than the other models (see Table 3), which shows that the four-factor measurement model has good discrimination validity.

**Table 3:** Confirmatory factor analysis result.

Model	*χ* ^2^	df	*χ*^2^/df	CFI	TLI	RMSEA
4-factor model a	628.232	454	1.384	0.916	0.922	0.045
3-factor model b	1181.476	457	2.584	0.876	0.881	0.067
3-factor model c	1226.252	457	2.683	0.865	0.873	0.071
2-factor model d	1388.211	459	3.024	0.775	0.785	0.093
Single factor model e	2546.886	460	5.535	0.693	0.627	0.157

^a^ In the 4-factor model, there is no relationship between all variables measured. Merging inclusive leadership and perceived insider status into a potential factor. ^C^ Merging supportive organizational climate and perceived insider status into a potential factor. ^d^ Merging inclusive leadership, perceived insider status, and perceived insider status into a potential factor. ^e^ Merging all variables into a potential factor.

### 4.4. Hypothesis tests

#### 4.4.1. Main effect and mediating effect

To test the main effect of IL on ER and the mediating effect of PIS, this study used a multilayer linear model approach to test the hypotheses, and the results are specified in Table 4. In the first step, after controlling for gender, age, education, and years of experience, the regression coefficient of inclusive leadership on employee psychological resilience was significantly positive and the main effect was verified (Model 6, *β* = 0.246, *p* < 0.01). To test the mediating role of PIS in the relationship between IL and EPR, the direct effect of inclusive leaders on PIS was tested by constructing Model 2 according to the procedure proposed by Baron and Kenny to test the mediating effect. As shown in Table 3, inclusive leaders had a significant positive effect on PIS (Model2, *β* = 0.598, p < 0.01). In the third step, constructing Model 7, the independent variable is put into both IL and mediating variable of PIS, and the dependent variable is put into EPR. The results show that the effect of IL is no longer significant (*β* = 0.127, ns), indicating that PIS has a fully mediating effect. Hypothesis 3 was verified.

**Table 4:** Hierarchical regression result.

Variable	PIS				EPR				
	Model 1	Model 2	Model 3	Model 4	Model 5	Model 6	Model 7	Model 8	Model 9
(Constant)	3.825**	1.159**	0.693	3.955	3.859	2.761	2.530	1.270	0.730
1.Gender	0.309**	0.168	0.121	0.104	0.145	0.088	0.054	−0.063	−0.060**
2.Age	−0.290**	−0.141	−0.164*	−0.176	−0.203**	−0.141	−0.113	−0.216**	−0.214
3.Education	−0.010	0.037	0.025	0.023	0.043	0.063	0.055	0.025	0.025
4.Tenure	0.029	0.047	0.045	0.040	0.044**	0.108	0.098*	0.102	0.102
5.IL		0.598**	0.542**	0.526**		0.246***	0.127	0.068	0.070
6.PIS			0.206**				0.200**		
7.SOC				−0.629**				0.659**	0.797**
8.IL*SOC				0.226**					−0.037
*R* ^2^	0.061	0.338	0.361	0.381	0.039	0.106	0.144	0.440	0.441
△*R*^2^	0.061	0.278	0.023	0.020	0.039	0.067	0.038	0.334	0.001
△*F*-value	3.472**	89.836**	7.582**	6.968**	2.163	16.161**	9.418**	126.920**	0.302

*n* = 220, **indicate *p* < 0.01. *Indicate *p* < 0.05.

To further test the robustness of the mediation effect, the results obtained by the Bootstrap method with the resampling set to 5,000 times showed a significant indirect effect of PIS (index = 0.119, CI = [0.06, 0.18]). This suggests that the mediation effect holds and hypothesis 3 is again supported by the data (see Table 5).

**Table 5:** Bootstrap analysis results of mediation effect.

Path	Coefficient	SE	BC (95% CI)
Total effect of IL on ER	0.246**	0.061	[0.125, 0.367]
Direct effect of IL on ER	0.126	0.071	[−0.014, 0.268]
Indirect effect of IL on ER (PIS as mediator)	0.119	0.004	[0.030, 0.213]

*p < 0.05; **p < 0.01; SE, CI are standard errors, confidence intervals of indirect effects estimated by bias-corrected percentile Bootstrap method.

### 4.5. Moderating effect

An evaluation of the moderating effect of SOC between PIS and EPR was conducted using hierarchical regression analysis. As can be seen from Table 3, in Model 4, after controlling for the main effect of IL and SOC on EPR, the interaction term of IL and SOC has a significant effect on EPR (Model4, *β* = 0.226, *p* < 0.01), indicating that The significant moderating effect of SOC between IL and EPR was verified by H4. In Model 6, after controlling the main effect of IL and SOC on EPR, the interaction items of IL and SOC have no significant effect on EPR (Model 9, *β* = −. 037, ns), indicating that the regulatory effect of SOC between IL and EPR is not significant, and H5 is not supported by data.

**Figure 2:**
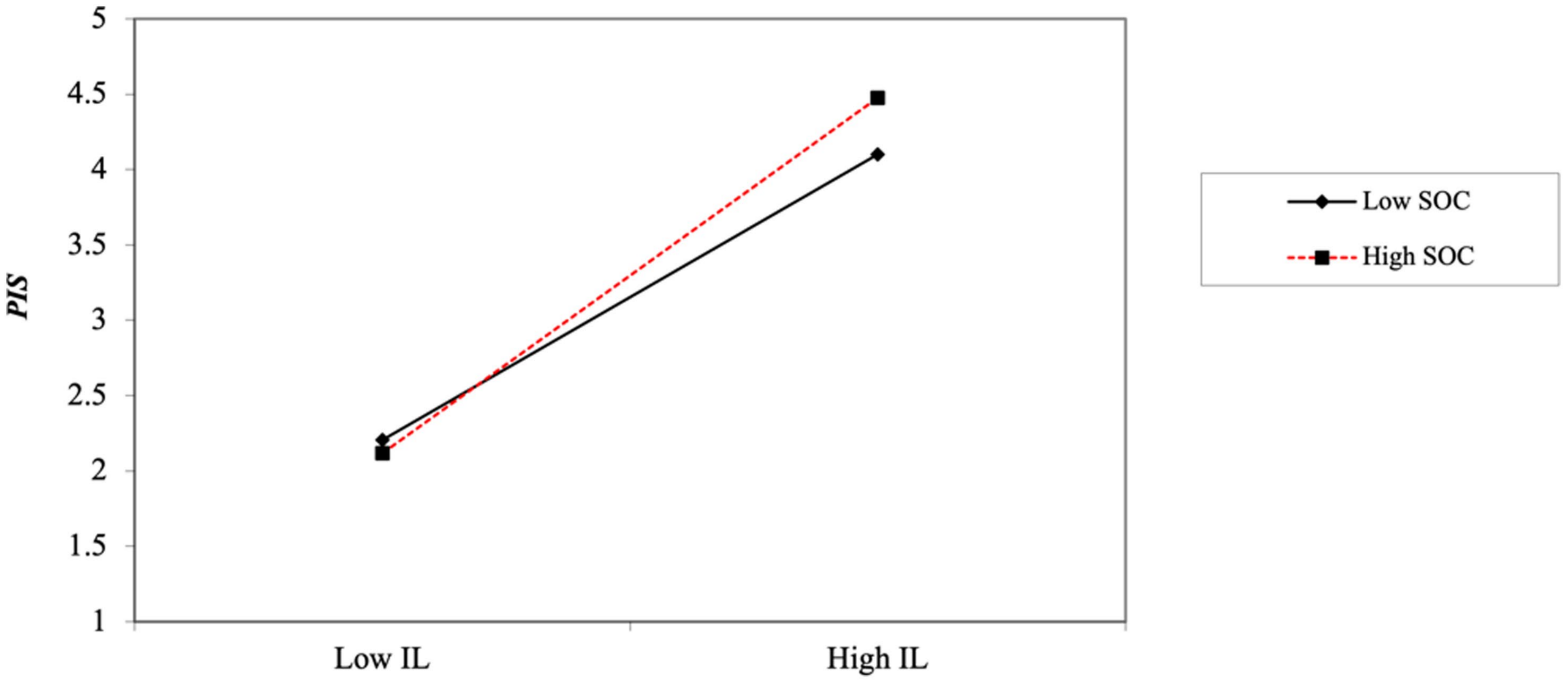
Hierarchical regression results.

This study also plots the moderating effect of SOC to further explain how SOC plays a moderating role in IL and EPR. The positive contribution of IL to EPR was stronger for employees with a higher perception of SOC compared to those with a lower perception of SOC (see Figure 2).

### 4.6. Moderated mediation

A moderated mediation model posits that the moderating variable would strengthen or weaken the relationship between the independent variable and the mediator variable, and at the same time strengthen or weaken the relationship between the mediator variable and the outcome (dependent) variable; as such, mediating effects would change due to the change of the moderating variable. Hypothesis 4 suggested that SOC would strengthen the mediating effect of PIS between IL and EPR. To test this hypothesis, this paper analyzed the mediating effect of PIS under different SCOs using the Bootstrapping Method with IL as the independent variable, as suggested by Edwards and Lambert (2007). The results of the analysis are shown in Table 6. The positive effect of Phase1 is significant (*β* = 0.216, p < 0.01), when the SOC is low, the positive effect of the first stage is significant (*β* = 0.409, p < 0.01), the positive effect of the first stage is also significant when the SOC is high (*β* = 0.710 p < 0.01). The influence of Phase 2 is not significant (*β* = − 0.073, ns). The regulatory role of SOC is mainly reflected in the path from PIS to EPR. In addition, Low SOC (*β* = 0.073, ns) and High SOC (*β* = − 0.028, ns) have no significant direct effect on ER in IL. SOC has a significant impact on indirect effects (index = 0.026, *p* < 0.05), and its CI values do not include 0 (CI = [0.003, 0.061]). SOC has a significant regulatory effect on the intermediary effect of PIS in the relationship between IL and EPR, that is, the regulatory intermediary effect of SOC is established, and H4 is verified.

**Table 6:** Bootstrap analysis results of Moderated Mediating Effect.

Conditional	Coefficient	SE	BC (95% CI)
	IL(X) → PIS(M)		
Phase1	0.216**	0.067	[0.085, 0.348]
Low-SOC(W)	0.409**	0.073	[0.264, 0.553]
High-SOC(W)	0.710**	0.075	[0.562, 0.859]
	PIS(M) → ER(Y)		
Phase2	−0.073	0.074	[−0.218, 0.072]
Direct effect	IL(X) → ER(Y)		
Low-SOC(W)	0.073	0.101	[−0.125, 0.273]
High-SOC(W)	−0.028	0.070	[−0.166, 0.109]
Indirect effect	IL(X) → PIS(M) → ER(Y)		
Low-SOC(W)	0.049	0.297	[0.005, 0.113]
High-SOC(W)	0.086	0.047	[0.010, 0.178]
moderated mediation index:	0.026	0.016	[0.003, 0.061]

n = 220; ** indicates *p* < 0.01, * indicates *p* < 0.05; Phase1 refers to the effect of IL on PIS, Phase 2 refers to the effect of PIS on employee psychological resilience, direct effect refers to the effect of IL on employee psychological resilience, and indirect effect refers to the product of Phase 1 and Phase 2.

## 5. Discussion

This study goes beyond past research on EPR in organizations. From the viewpoint of inclusive leaders and with the help of social identity theory and social information processing theory, we investigated the boundary conditions and process of inclusive leaders affecting EPR and constructed a moderated mediation model. We found evidence that IL directly influenced PIS. The finding is consistent with previous scholars’ assertions that PIS relates positively to altruism (Stamper and Masterson, 2002; Lapalme et al., 2009). Inclusive leaders emphasize two-way interactions with employees, respect and support them, and tend to explain the positive meaning of events so that employees make relatively optimistic evaluations, which in turn enhance their psychological resilience. Second, our results also indicate the importance of the PIS enhancing perceptions of a group membership. Inclusive leaders are open and inclusive, encouraging and appreciating the contributions of their subordinates, which encourages employees to feel recognized. If employees believe that their employer values them by treating them as inside members of the organization, they are more likely to exhibit higher psychological resilience. In addition, we argued that SOC would offer insights into the relationship between IL and EPR. Individuals may unconsciously self-adjust their identity in the organization based on the supportive climate they receive, which affects psychological resilience.

### 5.1. Theoretical significance

The theoretical contributions of this study are mainly as follows:

First, this study focused on how EPR is affected by inclusive leaders in the workplace. The results helped to enrich the existing literature on leadership styles and EPR by expanding the specific application of inclusion to resilience (Nguyen et al., 2016; Meng et al., 2019; Eliot, 2020). This provides a new perspective for studying EPR and enriches the exploration of the question “what factors promote employee psychological resilience.” Second, from the perspective of social identity, this paper explains the mechanism underlying the influence of inclusive leaders on EPR and confirms the mediating role played by the perception of insider identity, that is, the openness and tolerance of leaders and their concern for subordinates will make team members feel identified by the leaders and promote employees’ perception of insider identity, which will further enhance employees’ psychological resilience. This response to the call of scholars to expand the path of building resilience (Seville, 2018; Tonkin et al., 2018; Näswall et al., 2019; Zhu et al., 2019). Third, relying on social information processing theory, this paper integrates three elements of cues, environment, and information processing mechanisms and explores and validates the endogenous paths and boundaries of inclusive leaders’ interventions in EPR, using employee insider identity perception as a mediating variable and supportive organizational climate as the boundary of action responded to the call for an inquiry into the mechanism of the role of leadership type in influencing positive behavioral output (Rego et al., 2017; Eliot, 2020; Ahmed et al., 2021). The effectiveness of inclusive leaders and their transmission mechanisms are bound to vary across scenarios, and the moderated mediation model proposed in this paper, based on supportive organizational climate as a moderating variable, extends to some extent the validity of contextual factors in IL research and contributes to a deeper understanding of the effectiveness of inclusive leaders. Specifically, when the SOC is high, team members have a more positive perception of the leader and thus perceive themselves to be more aligned with the leader in terms of values matching, reinforcing the positive impact of insider perception of identity on EPR. Conversely, team members tend to feel isolated when the SOC is low and have difficulty gaining sufficient psychological capital from insider identity. This is consistent with the research conclusions of Caniëls et al. (2022) who argued that the perception of learning climate plays an essential role between trait resilience and resilient behavior. Overall, the findings of this study have important theoretical implications in that they suggest that regardless of whether team members perceive themselves to be insiders or outsiders in the overall team resource and power distribution if the team has a highly supportive climate, team members are less likely to have identity bias, which enhances their psychological resilience. The results also corroborate the view that high levels of organizational support can change employees’ perceptions of insider identity, leading to higher commitment and psychological perceptions and a greater tendency to exhibit high levels of psychological resilience. These results are consistent with the person-environment fit theory emphasizing the importance of organizational climate before coping with stressors (Caniëls et al., 2022). There is a lack of research exploring and testing the role and position of SOC in the process of inclusive leaders affecting EPR, so this study can be considered a helpful extension and supplement to previous research.

### 5.2. Practical significance

This study also has important implications for the management practices of organizations. This study focused on psychological resilience during the COVID-19 pandemic. Since the impact of the COVID-19 pandemic on psychological resilience remains unpredictable and little is known, enhancing resilience and coping strategies should be helpful for employees’ psychological health, job performance and well-being. Psychological resilience is indispensable as an essential behavioral competency for organizational employees in daily work and extraordinary situations (Tonkin et al., 2018), and we believe that organizational EPR can be enhanced in various ways, including leadership style, organizational climate, and psychological resources. First, this study shows that inclusive leaders have a direct and significant positive impact on the psychological resilience of team members. Therefore, managers should strengthen inclusive leaders’ behavioral capabilities and create an inclusive learning organizational climate. Inclusive leaders help employees be open to a diversity of information through a social penetration process (Owens and Hekman, 2016) and convey positive behavioral concepts through role cognition. Organizations should focus on leadership development and training programs to help leaders recognize the importance of inclusion and develop it. inclusive leaders’ behavior is an ongoing process that depends heavily on the leaders’ knowledge, experience, and skills. Accordingly, exceptional training programs should be strengthened to focus on the development of leaders’ professionalism and personal competencies so that the externalized inclusive behaviors (acknowledging shortcomings and mistakes, evaluating oneself objectively, appreciating employees’ contributions and strengths, being open to new ideas and actively seeking feedback) can gradually be solidified as intrinsic qualities of leaders.

Second, inclusive leaders contribute to employees’ interpretation of high-quality supervisory relationships (Owens and Hekman, 2016) and play an important role in motivating employees at work. Managers should focus on cultivating and strengthening employees’ insider identity perceptions. In the management process, leaders should be open and inclusive, respect individual employees and other behaviors, strengthen more interpersonal interactions with team members through formal or informal channels, and improve communication and understanding between subordinates and superiors, which helps to enhance subordinates’ perceptions of their insider identity, improves employees’ work well-being and mental toughness, and triggers employees to motivation, initiative, and adaptive behavior in response to extraordinary circumstances (e.g., organizational crises).

In addition, an individual’s perception of organizational support affects the individual’s cognitive judgment of his or her identity or status in the organization and the individual’s evaluation of self, ultimately affecting the individual’s psychological resilience. Organizations need to create a supportive organizational climate, for example, by creating a relaxed and free soft environment, encouraging free speech, providing opportunities for employees to express their opinions, valuing employees’ ideas, and appropriate leadership empowerment. By developing positive human resource management strategies, respecting, and recognizing employees from top to bottom, meeting the emotional needs of employees to become “insiders” of the organization, and improving employees’ sense of identity and belonging to the organization, we can better promote the improvement of employees’ psychological resilience and maximize the effectiveness of managers’ inclusive leaders.

### 5.3. Research limitations and directions of future research

This paper has certain research limitations, which also provide insights for future research. First, from the perspective of research design, although this study collects data from two-time points, the predictor variable (perceived insider status) and dependent variable (employee psychological resistance) in the research framework collect data at the same time point, which is not conducive to inferring the causal relationship of variables. With the time-lags design, this research strengthens the causal inference, but our conclusions should be interpreted cautiously yet. More research is needed to verify further the causal relationship among those variables with experiments or multi-time point longitudinal survey design (i.e., three-time points). In addition, due to the limited sample size and type of companies investigated in this study, it is questionable whether the effect of inclusive leadership on employee psychological resilience can be similarly generalized to other samples. Future studies may try to expand the sampling scope of the study, strive to select samples across cultures and borders, and appropriately increase the sample size to improve the persuasiveness and generalizability of the findings.

Second, this study is based on the individual perspective to explore the impact mechanism of IL on EPR. Although the data analysis results support our hypothesis, it be noted that like other human psychology and behavior, psychological resilience should also be a function of the individual and environment. Subsequent research can further introduce organizational situational variables (e.g., learning climate, team interactions) to explore the mechanism of interaction between individuals and situational variables on psychological resilience. Moreover, individual differences may affect PIS and psychological resilience. Future research could incorporate individual differences and diversity concepts to create a richer and more three-dimensional research framework.

Third, cultural variability across countries may still cause some bias. The moderating effects of power variables such as collectivism and power distance can also be incorporated into future studies, which may lead to richer findings.

## 6. Conclusion

This study provides a new research perspective and empirical study of the relationship between inclusive leadership and employee psychological resilience by constructing a moderated mediation model, which is a useful addition to the employee psychological resilience literature and helps us better understand the impact of inclusive leadership on employee psychological resilience. The results confirm that inclusive leadership enhances employees’ psychological resilience in the workplace. Employee insider identity perception mediates the relationship between inclusive leadership and employee psychological resilience; supportive organizational climate moderates the indirect effect of inclusive leadership on employee psychological resilience through employee insider identity perception, i.e.: Contexts with high (compared to low) supportive organizational climate make it easier for inclusive leaders to stimulate employees’ insider identity perception, which positively affects employee psychological resilience.

## Data availability statement

The raw data supporting the conclusions of this article will be made available by the authors, without undue reservation.

## Author contributions

PP contributed to conception and design of the study. XL organized the database and performed the statistical analysis. PP and XL wrote the first draft of the manuscript. All authors contributed to the article and approved the submitted version.

## Funding

This work was supported by Shandong Jianzhu University Ph.D. Fund Project (XNBS643) and Qingdao Social Science Planning Project (QDSKL2201204).

## Conflict of interest

The authors declare that the research was conducted in the absence of any commercial or financial relationships that could be construed as a potential conflict of interest.

## Publisher’s note

All claims expressed in this article are solely those of the authors and do not necessarily represent those of their affiliated organizations, or those of the publisher, the editors and the reviewers. Any product that may be evaluated in this article, or claim that may be made by its manufacturer, is not guaranteed or endorsed by the publisher.

## Appendix A

**Table:**

Items used to measure the study variables	Factor loadings	CR	AVE
Inclusive leadership (Alpha = 0.896)		0.930	0.598
The manager is open to hearing new ideas (Openness)	0.813		
The manager is attentive to new opportunities to improve work processes (Openness)	0.805		
The manager is open to discuss the desired goals and new ways to achieve them (Openness)	0.807		
The manager is available for consultation on problems (availability)	0.725		
The manager is an ongoing ‘presence’ in this team-someone who is readily available (availability)	0.700		
The manager is available for professional questions I would like to consult with him/her (availability)	0.852		
The manager is ready to listen to my requests (availability)	0.785		
The manager encourages me to access him/her on emerging issues(accessibility)	0.771		
The manager is accessible for discussing emerging problems(accessibility)	0.684		
Perceived insider status (Alpha = 0.856)		0.909	0.627
I feel very much a part of work organization	0.766		
My work organization makes me believe that I am included in it	0.808		
I feel like I am an “outside” at this organization(R)	0.756		
I do not feel included in this organization(R)	0.823		
I feel I am an “inside” in my work organization	0.811		
My work organization makes me frequently feel ‘left-out’(R)	0.785		
Supportive Organizational Climate (Alpha = 0.838)		0.881	0.597
Managers consistently treat everyone with respect	0.766		
Managers clearly communicate work objectives and responsibilities	0.808		
Managers take action on new ideas provided by employees	0.756		
Departments cooperate to get the job done effectively and efficiently	0.771		
I have a good working relationship with my manager	0.763		
Psychological resilience (Alpha = 0.938)		0.948	0.669
I effectively collaborate with others to handle unexpected challenges at work	0.750		
I successfully manage a high workload for long periods of time	0.734		
I resolve crises competently at work	0.852		
I learn from mistakes at work and improve the way I do my job	0.867		
I re-evaluate my performance and continually improve the way I do my work	0.873		
I effectively respond to feedback at work, even criticism	0.798		
I seek assistance to work when I need specific resources	0.811		
I approach managers when I need their support	0.852		
I use change at work as an opportunity for growth	0.816		
